# Genome-wide identification, phylogenetic analysis, and expression profiles of trihelix transcription factor family genes in quinoa (*Chenopodium quinoa* Willd.) under abiotic stress conditions

**DOI:** 10.1186/s12864-022-08726-y

**Published:** 2022-07-10

**Authors:** Kuiyin Li, Yue Fan, Guangyi Zhou, Xiaojuan Liu, Songshu Chen, Xiangcai Chang, Wenqiang Wu, Lili Duan, Maoxing Yao, Rui Wang, Zili Wang, Mingfang Yang, Yanqing Ding, Mingjian Ren, Yu Fan, Liyi Zhang

**Affiliations:** 1 College of Agriculture, Guizhou University, Huaxi District, Guiyang City, Guizhou Province 550025 P.R. China; 2 College of Agriculture, Anshun University, Anshun, 561000 P.R. China; 3 College of Food Science and Engineering, Xinjiang Institute of Technology, Aksu, 843100 P.R. China; 4 Institute of Upland Food Crops, Guizhou Academy of Agricultural Sciences, Huaxi District, Guiyang City, Guizhou Province 550006 P.R. China; 5 Guizhou Branch of National Wheat Improvement Center of Guizhou University, Guiyang, 550025 P.R. China

**Keywords:** *Chenopodium quinoa* Willd., Trihelix genes, Abiotic stress, Gene duplication

## Abstract

**Background:**

The trihelix family of transcription factors plays essential roles in the growth, development, and abiotic stress response of plants. Although several studies have been performed on the trihelix gene family in several dicots and monocots, this gene family is yet to be studied in *Chenopodium quinoa* (quinoa).

**Results:**

In this study, 47 *C. quinoa* trihelix (CqTH) genes were in the quinoa genome. Phylogenetic analysis of the CqTH and trihelix genes from *Arabidopsis thaliana* and *Beta vulgaris* revealed that the genes were clustered into five subfamilies: SIP1, GTγ, GT1, GT2, and SH4. Additionally, synteny analysis revealed that the CqTH genes were located on 17 chromosomes, with the exception of chromosomes 8 and 11, and 23 pairs of segmental duplication genes were detected. Furthermore, expression patterns of 10 CqTH genes in different plant tissues and at different developmental stages under abiotic stress and phytohormone treatment were examined. Among the 10 genes, *CqTH02*, *CqTH25*, *CqTH18*, *CqTH19*, *CqTH25*, *CqTH31*, and *CqTH36*, were highly expressed in unripe achenes 21 d after flowering and in mature achenes compared with other plant tissues. Notably, the 10 CqTH genes were upregulated in UV-treated leaves, whereas *CqTH36* was consistently upregulated in the leaves under all abiotic stress conditions.

**Conclusions:**

The findings of this study suggest that gene duplication could be a major driver of trihelix gene evolution in quinoa. These findings could serve as a basis for future studies on the roles of CqTH transcription factors and present potential genetic markers for breeding stress-resistant and high-yielding quinoa varieties.

**Supplementary Information:**

The online version contains supplementary material available at 10.1186/s12864-022-08726-y.

## Background

In plants, more than 60 transcription factor (TF) families have been discovered, which play critical roles in the growth, development, and abiotic stress responses of various plant species [1–3]. In the 1980s, trihelix TFs were isolated for the first time in pea (*Pisum sativum*), and were found to occur solely in plants [4]. Trihelix TFs typically attach to the core sequence (5′-G-Pu-[T/A]-A-[T/A]-3) of ribulose-1,5-bisphosphate carboxylase-3A (*rbcS-3A*) promoter region to regulate light-dependent expression [5]. Additionally, the trihelix structure of GT factors is similar to that of Myb/SANT-LIKE DNA-binding domains [6]. However, the gaps between helix pairs result in distinct recognition sequences between GT factors and Myb/SANT-like proteins [6, 7].

Over the years, the trihelix family of TFs has been extensively studied in both dicots and monocots, including *Arabidopsis thaliana*, *Solanum lycopersicum*, *Chrysanthemum × morifolium*, *Glycine max*, *Triticum*, *Zea mays*, *Oryza sativa*, *Fagopyrum esculentum,* and *Sorghum bicolor*. Extensive studies have been performed on the trihelix gene family in different species under different stress conditions and at a different developmental stage owing to their role in plant development and environmental adaptation. For instance, a total of 30 GT members have been identified in *A. thaliana*, and divided into subfamilies, including GT1, GT2, GTγ, SH4, and SIP1 [8]. Additionally, 36 trihelix proteins have been identified in tomato, and divided into the GT1, GT2, SH4, SIP1, GTγ, and GTδ subfamilies [9]. Interestingly, the structures of most trihelix genes vary among plant species, especially at the C-terminal.

Furthermore, trihelix genes play an intricate physiological role in plants. For instance, ectopic expression of *TaGT2L1D* influences floral organ development and growth in wheat [10]. Additionally, *A. thaliana asil1* mutant seedlings exhibited changes in gene expression profile, which was similar to the expression during late embryogenesis [8]. Moreover, *GT2-like 1* (*GTL1*) and its homolog *DF1* inhibit root hair growth by directly binding to and regulating the expression of *ROOT HAIR DEFECTIVE SIX-LIKE4* (*RSL4*) activator. Loss of function of nuclear *GTL1* during the post-branching phase of trichome development can increase the nuclear DNA content of trichomes that have completed branching [11]. Furthermore, the role of trihelix gene family in abiotic stresses has been examined. For instance, exposure to light enhanced the expression of GT1 subfamily genes, which are possibly involved in salt stress and pathogen infection responses, in 3-day-old *A. thaliana* seedlings [12]. Additionally, exposure to light inhibited the expression of the *GT1* gene *RML1* in yellow seedlings of *S. lycopersicum* [13]. Moreover, the trihelix TFs *GmGT-2A* and *GmGT-2B*, were activated in soybeans under osmotic, salt, and cold stress conditions [14]. Interestingly, *GTL1* mutations can reduce transpiration considerably and increase drought tolerance in *A. thaliana* [15]. Moreover, there was a 2.5–10-fold increase in the expression of the GT evolution branch gene *OsGT-1* in tomatoes in response to salt stress and abscisic acid (ABA) exposure [16]. However, the functions of trihelix genes in signal transduction pathways associated with various stress responses requires further investigation.

Quinoa (*Chenopodium quinoa* Willd.) is a 5000-year-old plant with seeds rich in nutrients and bioactive compounds. Quinoa can be grown at altitudes ranging from sea level to 4500 m on high plateaus owing to its drought, cold, and salt tolerance [17]. However, there is limited information on the trihelix family genes in quinoa.

Therefore, the aim of this study was to identify and characterize trihelix genes in quinoa. Specifically, the chromosomal locations, protein properties, gene architecture, and conserved motif compositions of the identified trihelix genes were analyzed. The orthologous relationships and gene duplication events among the trihelix genes were also examined. Additionally, we investigated the expression patterns of selected genes in different tissues, under different abiotic stresses, at different periods after flowering, and at different periods after phytohormone treatments.

## Results

### Trihelix genes in *C. quinoa* and their physicochemical properties

A total of 47 non-redundant trihelix genes were identified in *C. quinoa*, and the genes were designated Cqtrihelix1–Cqtrihelix47 (*CqTH1*–*CqTH47*) based on their chromosomal positions, and their physicochemical properties are listed in Table S1. *CqTH38* encodes the smallest protein with 194 amino acids, whereas *CqTH24* encodes the largest protein with 826 amino acids. The molecular weight of the CqTH proteins ranged from 21.54–94.44 kDa, while the predicted isoelectric points ranged from 4.37 (*CqTH12*) to 10.31 (*CqTH42*). Subcellular localization analysis showed that 34 genes were located in the nucleus, 6 in the cytoplasm, 4 in the chloroplast, and 1 each in the plasmids, mitochondria, and extracellular membrane. Among the 47 genes, two genes (4.26%; *CqTH28,* and *CqTH31*) contained the GT1 superfamily domain (4.26%), whereas the remaining 45 genes (95.74%) contained the Myb/SANT-like DNA-binding domain. Additionally, the CqTH genes constitutes 0.11% of the total genes in *C. quinoa* genome [18].

### Phylogenetic relationships between the trihelix genes in *C. quinoa* and other plants

**Fig. 1:**
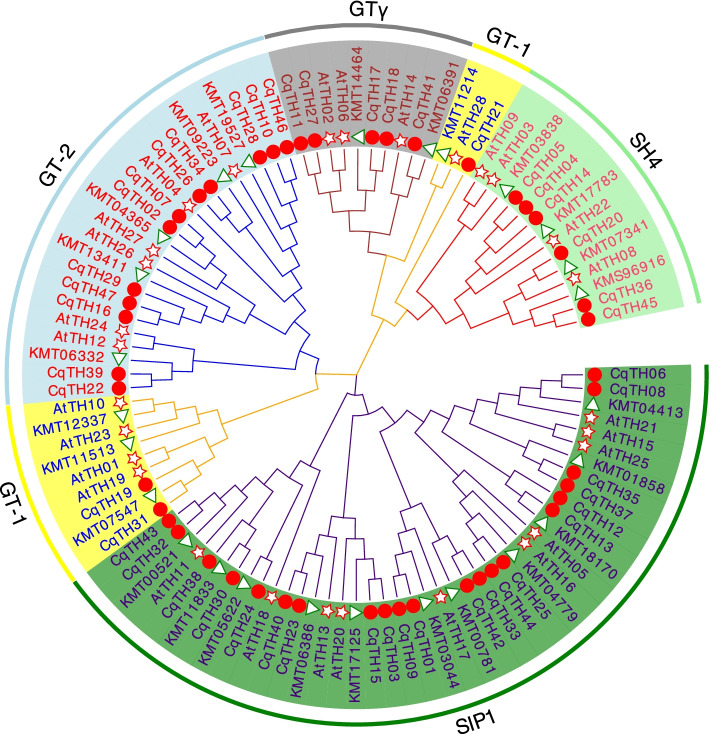
The phylogenetic tree was derived using the NJ method in MEGA7. Unrooted phylogenetic tree showing relationships among trihelix domains of *C. quinoa* (red solid circle), *A. thaliana* (red star) and *B. vulgaris* (green triangle)*.* As shown in the figure, the phylogenetic tree is divided into 5 subfamilies, including subfamily SIP1, GTγ, GT1, GT2, and SH4

To understand the relationship between trihelix genes, we constructed a phylogenetic tree using the amino acid sequences of 47 CqTH genes, 28 *A. thaliana* trihelix (AtTH), and 26 *B. vulgaris* trihelix (BvTH) proteins using the neighbor-joining (NJ) method of MEGA 7.0 with a bootstrap value of 1000 (Fig. 1 and Table S1). According to the topological tree structure and classification approach by Kaplan-Levy et al. [19] and Qin [20], the 47 CqTH genes were clustered into five groups: SIP1, GTγ, GT1, GT2, and SH4, which were analogous to those in *A. thaliana* and *O. sativa*. Specifically, the SIP1, GTγ, GT1, GT2, and SH4 subfamilies contained 21, 5, 3, 11, and 7 genes, respectively. Although we detected 23 gene duplication events in the quinoa genome, there were no differences in the sequences of the 47 trihelix proteins shared among the three species during evolution.

### Motif compositions and exon/intron structures of the trihelix genes in *C. quinoa*

**Fig. 2:**
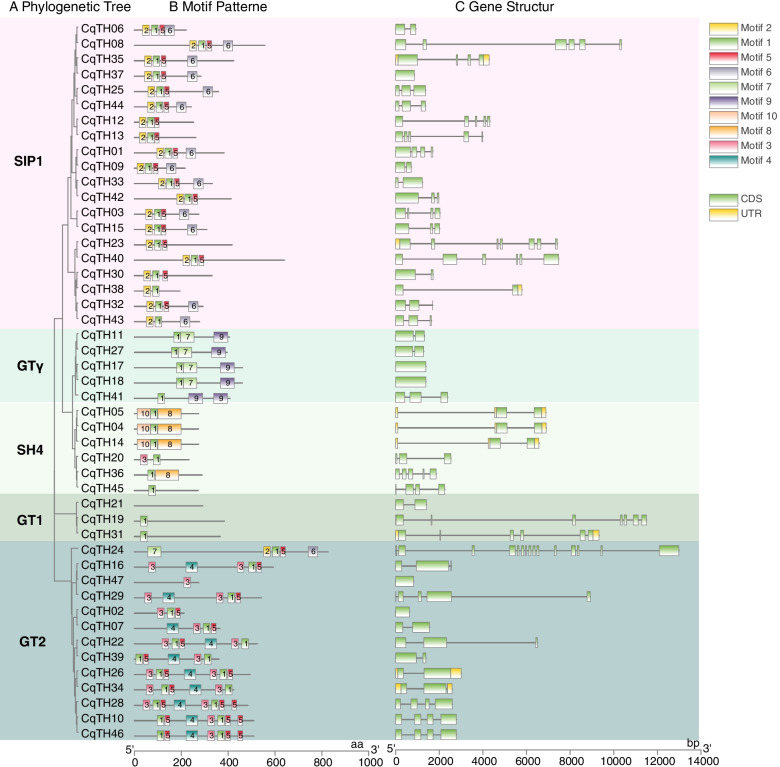
Phylogenetic relationships, gene-structure analysis, and motif distributions of CqTH trihelix genes. **A** Phylogenetic tree was constructed based on the full-length sequences of CqTH proteins using the NJ method with 1000 replicates on each node, including group SIP1, GTγ, GT1, GT2, and SH4. **B** The motif composition of the CqTH proteins. The motifs, numbered 1–10, are displayed in different colored boxes. The sequence information for each motif is provided in Additional file 2. The length of the protein can be estimated using the scale at the bottom. **C** Exon-intron structures of CqTH genes. Yellow boxes indicate untranslated 5′- and 3′- regions; Blue-green boxes indicate exons; and black lines indicate introns

The MEME search tool (http://meme.nbcr.net/meme/intro.html) was used to predict 10 conserved motifs in CqTH genes, which were designated as motifs 1–10. The 10 motifs were divided into 5 groups (SIP1, GTγ, GT1, GT2, and SH4), according to the similarity of motif pattern and gene structure (Fig. 2A). Additionally, the lengths of the conserved motifs ranged from 15 to 50 amino acid residues (Table S2). The motif arrangement of each CqTH protein is depicted with the matching color boxes in Fig. 2B. Motif 1 was observed in almost all CqTH proteins, while other groups shared comparable motifs, indicating that the conserved motifs may play a critical role in specific processes. In the GT2 subfamily, several genes contained more than one copy each of motifs 1, 3, or 5. Specifically, *CqTH16*, *CqTH22*, *CqTH26*, *CqTH28*, *CqTH29*, and *CqTH34* had two copies of motif 3; *CqTH10*, *CqTH22*, *CqTH26*, *CqTH28*, *CqTH34*, *CqTH39*, and *CqTH46* possessed two copies of motif 1; and *CqTH10, CqTH28,* and *CqTH46* contained three copies of motif 5. This may be considered a distinguishing feature between different subfamilies, and the different motif arrangements in some subfamilies could be attributed to structural differences in the amino acid sequences. Motifs 1, 2, 5, and 6 were detected in the majority of SIP1 members. However, *CqTH12*, *CqTH13*, *CqTH23*, *CqTH30*, *CqTH40*, and *CqTH42* did not contain motif 6, *CqTH38* did not contain motifs 5 and 6, and *CqTH43* did not possess motif 5. SH4 members mainly contained motifs 1, 8, and 10, but *CqTH20*, *CqTH36*, and *CqTH45* did not contain motif 10. Furthermore, *CqTH45* possessed only motif 1, which was similar to *CqTH19* and *CqTH31* in the GT1 subfamily. Notably, no motif was detected in the *CqTH21* gene of the GT1 subfamily. Motifs 1, 7, and 9 were observed in the GTγ subfamily members, with *CqTH24* containing two copies of motif 9 and no motif 7.

The exon/intron structures and phases were identified by mapping the full-length cDNAs of CqTH genes to the genome sequence. Members of the same family exhibited comparable exon/intron arrangement depending on the exon/intron number (Fig. 2C). The structural features of CqTH genes, including the number and placement of exons and introns, are depicted in Fig. 2C. Additionally, the introns isolated the coding sequences in the majority of trihelix genes (42, 89%). Structural analysis showed that no introns were present in five CqTH genes (*CqTH02/17/18/37/47*) (11%), whereas the highest number of introns was present in *CqTH24* (16). The number of exons in CqTH genes ranged from 1 to 15, of which the SIP1, GT2, SH4, GTγ, and GT1 subfamily members possessed 1–15, 1–5, 2–5, 1–3, and 4–7 exons, respectively. The GTγ subfamily had the fewest exons, while the GT1 family had the most.

### Cis-acting elements in the promoter region of CqTH gene family

Cis-acting elements serve as a molecular switch by binding to transcription factors, which are associated with gene transcription initiation and activity. To explore the putative functions of CqTH genes, we extracted and examined the 1500 bp sequences upstream of the transcription start site to identify cis-acting elements using PlantCARE Online program. Ten types of cis-acting elements were present in the promoter regions, including abscisic acid-, methyl jasmonate (MeJA)-, light-, gibberellin-, low-temperature-, and salicylic acid-, defense and stress-, and auxin-responsiveness elements, drought-inducibility element, and enhancer-like element involved in anoxic specific inducibility. The distribution of the cis-acting elements on the promoters is shown in Fig. S1. In terms of the number of cis-acting elements, *CqTH23* contains the most (23), while *CqTH40* contains the least (2). In terms of the number of types of cis-acting elements, *CqTH35* has the most types (9), while *CqTH20*, *CqTH26*, and *CqTH27* have only 1 type. Light responsive elements were present in almost all promoter regions of CqTH genes, with large numbers in *CqTH12* and *CqTH23*. A total of 33 CqTH genes contained abscisic acid-responsiveness element, 25 contained MeJA-responsiveness element, 21 contained low-temperature-responsiveness element, 16 contained salicylic acid-responsiveness element, 15 contained drought-inducibility element, and 14 contained defense and stress-responsiveness element. These results suggest that the CqTH genes in *C. quinoa* contain several environmental stress elements, which could play important roles in stress resistance.

### Chromosome distribution and synteny of the trihelix genes in *C. quinoa*

**Fig. 3:**
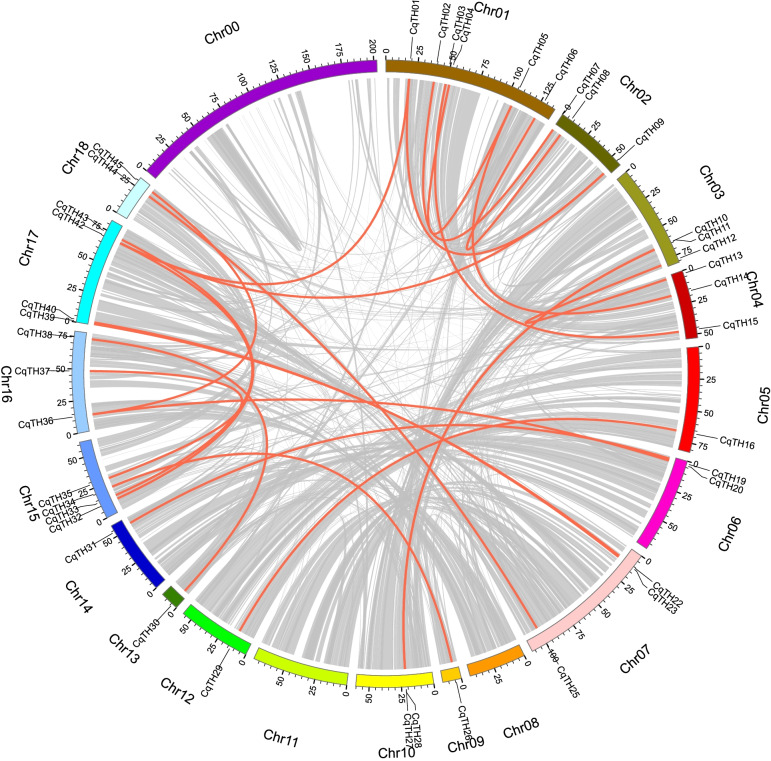
Schematic representation of the chromosomal distribution and interchromosomal relationships of CqTH genes. Different color arcs represent different chromosomes and the arc length represents the length of the chromosome. Chromosome number is indicated at the bottom of each chromosome. The red lines indicate duplicated trihelix gene pairs

The chromosome positions of CqTH genes were extracted from the genome annotation files. They were unevenly dispersed and non-randomly distributed at specific positions on chromosomes 1–18 (Chr01–Chr18) and Chr00, which was the remaining unassembled fragment in the quinoa genome (Fig. S2). The 47 CqTH genes were designated according to their physical locations on the *C. quinoa* chromosomes, from top to bottom. Chr01 has the highest number of CqTH genes (6, 12.8%), followed by Chr17 (5, 10.6%), Chr07 and Chr15 (4 genes each, 8.5%), Chr02, Chr03, Chr04, Chr05, Chr06, and Chr16 (3 genes each, 6.4%); Chr10, Chr18, and Chr00 (2 genes each, 4.3%); and Chr09, Chr12, Chr13, and Chr14 (1 gene each, 2.1%). Additionally, 23 pairs of segmental duplications were detected on the chromosomes (Fig. 3 and Table S3). A tandem duplication event is defined as a 200-kb chromosomal area with two or more identical genomic regions.

The CqTH gene family has 40 paralogs (85.1%), suggesting the evolutionary link between the members (Fig. 3). Additionally, the CqTH genes were unevenly distributed among 18 *C. quinoa* linkage groups (LGs). There were no LGs in Chr08 and Chr11, where the trihelix gene family was not detected, and in the unassembled Chr00. Certain LGs contained more CqTH genes than others, such as LG1 with six CqTH genes. Further analysis revealed that all genes were linked within their subfamilies. The SIP1 subfamily has the most related genes (20), accounting for half of the trihelix gene family’s linked genes, while the GT2 subfamily had nine linked genes.

### Evolutionary relationship between the trihelix genes in *C. quinoa* and other plants

An unrooted NJ tree with 10 conserved motifs was generated using Geneious R11 to identify the evolutionary relationship of the trihelix gene family between *C. quinoa* and other plants (i.e., *S. bicolor*, *O. sativa*, *B. rapa*, *S. lycopersicum*, *A. thaliana*, and *S. tuberosum*) (Fig. S3, Tables S2 and S4). The CqTH proteins in the evolutionary tree were relatively dispersed, with motifs 1 and 5 shared by multiple trihelix family members from various species (Fig. S3). Trihelix proteins from the same subfamily had similar motif compositions. Notably, similar serial motifs tended to cluster in *C. quinoa*, tomato, and potato, indicating that CqTH proteins may be more closely related to those of tomato and potato than those of the other plants.

**Fig. 4:**
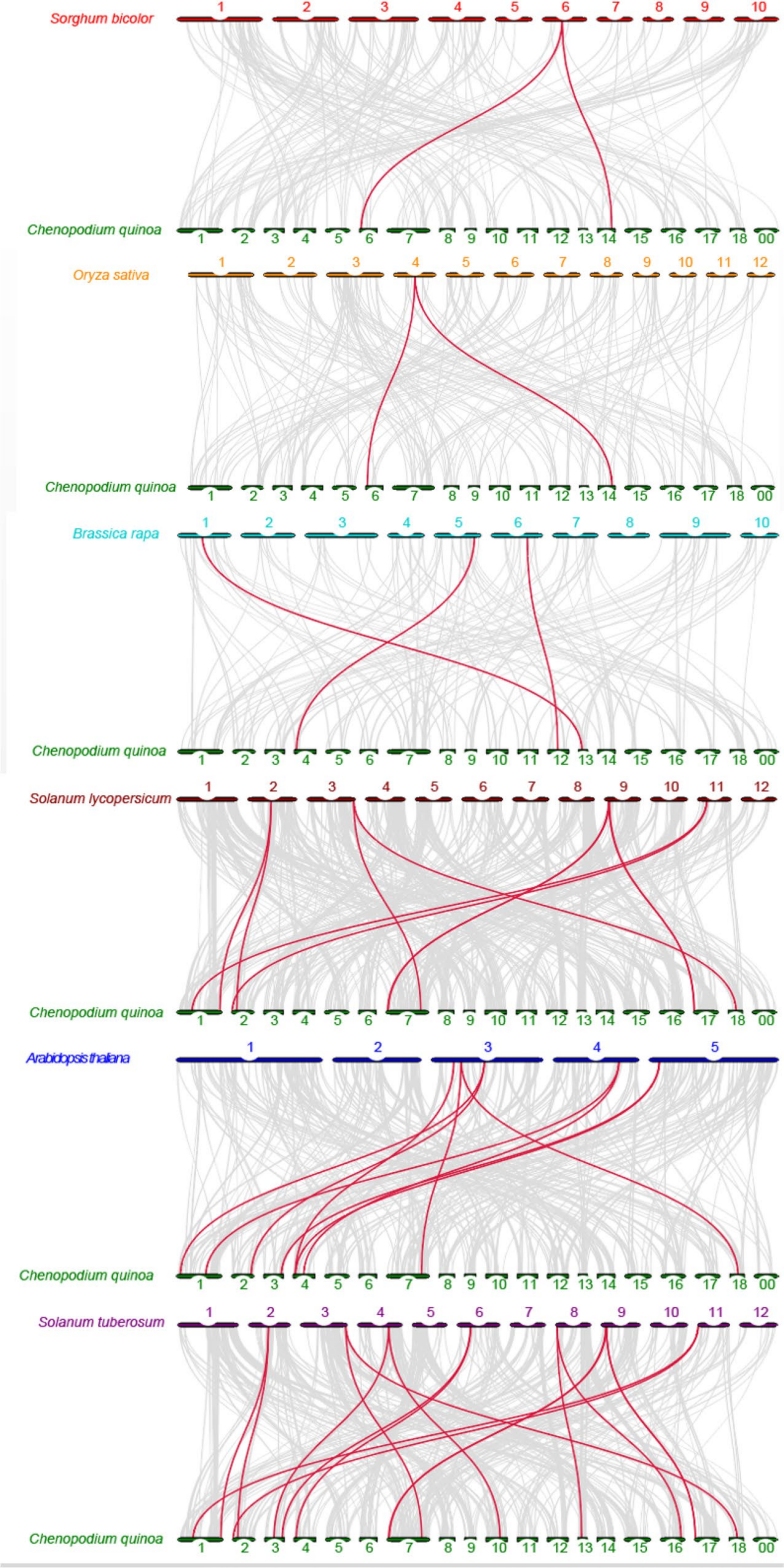
Synteny analyses of the trihelix genes between *C. quinoa* and six representative plant species (*S. bicolor*, *O. sativa*, *B. rapa*, *S. lycopersicum*, *A. thaliana*, and *S. tuberosum*). Gray lines in the background indicate adjacent blocks in the genomes of *C. quinoa* and six other representative plants, and red lines highlight gene pairs formed on the genomes of CqTH genes and six other representative plants

To investigate the gene replication mechanisms in *C. quinoa*, we constructed seven comparison system diagrams between *C. quinoa* and six species, including four dicots (*B. rapa*, *S. lycopersicum*, *A. thaliana*, and *S. tuberosum*) and two monocots (*S. bicolor*, *O. sativa*) (Fig. 4). The number of colinear genes between *C. quinoa*, *S. bicolor*, *O. sativa*, *B. rapa*, *S. lycopersicum*, *A. thaliana*, and *S. tuberosum* formed 2, 2, 3, 10, 9, and 16 homologous gene pairs, respectively (Table S4). Analysis of the system diagrams showed that quinoa was most similar to potato and least similar to sorghum and rice, which may indicate the evolutionary relationships among the species. Notably, 22 CqTH genes were unique to dicots, indicating that these genes may have evolved after the differentiation of dicotyledonous plants. Several CqTH genes were also associated with three synonymous gene pairs, including *CqTH13, CqTH25,* and *CqTH44*, which potentially played key roles in the trihelix gene family during evolution. Tajima’s D neutrality test was performed to determine the evolutionary role of the CqTH gene family, and the Tajima’s D value was − 0.875727 (Table S5), indicating that this gene family was strongly selected during the evolution of *C. quinoa*.

### Expression patterns of CqTH genes in different organs

**Fig. 5:**
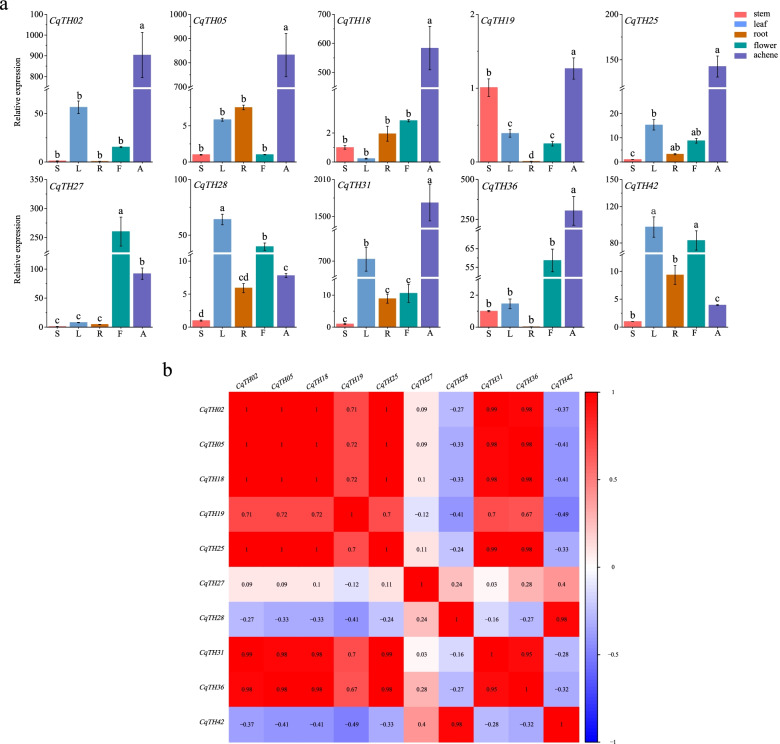
**a** Tissue-specific gene expression patterns of 10 CqTH genes in the root (R), stem (S), leaf (L), flower (F) and Achene (A) tissues were examined by qPCR. Error bars were obtained from three measurements. Lowercase letter(s) above the bars indicate significant differences (*p* < 0.05, LSD) among the treatments. **b** The correlation between the gene expression patterns of 10 CqTH genes. Red squares or positive number: positive correlation, light blue squares or negative number: negative correlation. The significance level was 0.01

The functional evaluation of various genes in plants revealed that trihelix genes play significant roles in crop growth and development [19]. The expression levels of 10 selected genes in different plant tissues were examined to determine the physiological role of CqTH genes in the growth and development of *C. quinoa*. Histograms were generated to depict the expression patterns of the CqTH genes in tissues, including roots, stems, leaves, flowers, and achenes (Fig. 5a, Table S6a). Notably, the CqTH genes were significantly expressed in specific tissues, suggesting that CqTH genes perform various functions in *C. quinoa* growth and development. Specifically, seven genes, including *CqTH02, CqTH05, CqTH18, CqTH19, CqTH25, CqTH31,* and *CqTH36*, were highly expressed in the achenes. Additionally, *CqTH28* and *CqTH42* were highly expressed in the leaves, whereas *CqTH27* was highly expressed in the flowers. Notably, *CqTH31* expression was highest in the achenes, whereas *CqTH42* expression was highest in the leaves. In contrast, the expression patterns of the 10 CqTH genes in the roots and stems of *C. quinoa* seedlings were low.

Additionally, we explored the correlation between the CqTH expression profiles and found that the majority of the CqTH genes were positively associated, particularly those that were highly correlated with several other genes (Fig. 5b). In contrast, *CqTH28* and *CqTH42* were negatively correlated with seven CqTH genes. Additionally, *CqTH27* was negatively associated with *CqTH19* and not associated with the remaining eight genes. However, *CqTH02*, *CqTH05*, *CqTH18*, and *CqTH25* were significantly positively correlated.

### CqTH gene expression patterns in response to diverse abiotic stresses

**Fig. 6:**
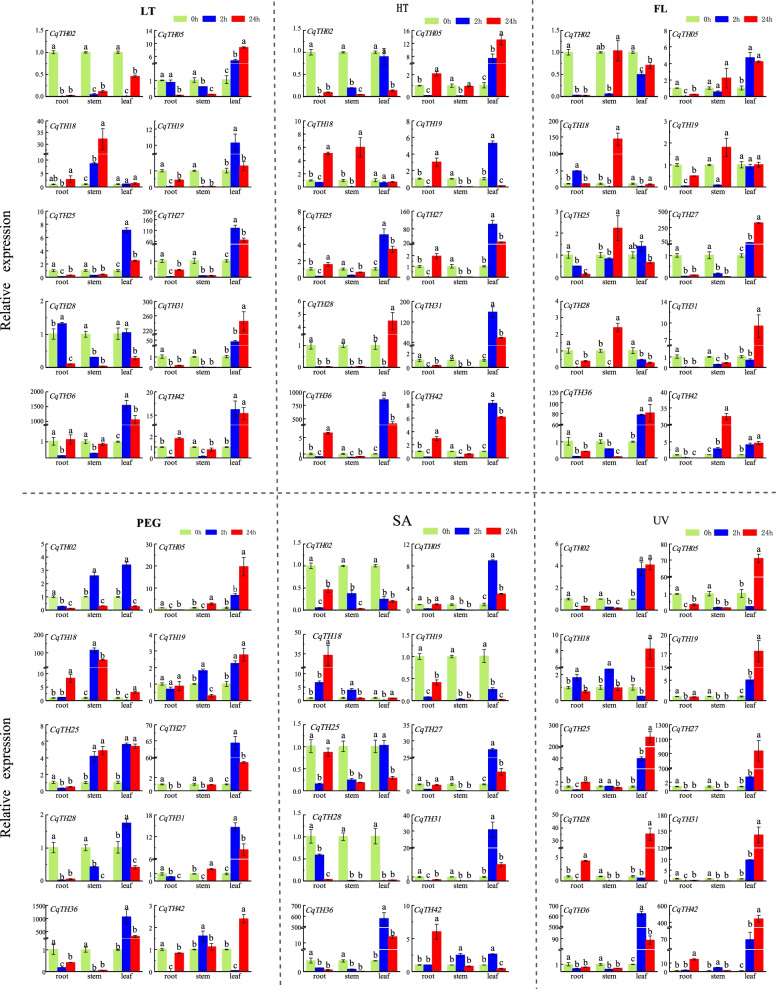
The expression patterns of 12 CqTH genes in roots, stems and leaves of quinoa seedlings treated with 2 h and 24 h under different abiotic stress (LT: Low temperature, HT: High temperature, FL: Flooding, PEG: Osmotic, SA: Salt, UV: Ultraviolet radiation) were examined by qPCR. Error bars were obtained from three measurements. Lowercase letter(s) above the bar indicates significant difference (*p* < 0.05, LSD) among the treatments

Furthermore, the expression profiles of 10 CqTH genes in response to diverse abiotic stress conditions, including high temperature, low temperature, osmotic pressure, flooding, salt, and UV radiation, were determined by qPCR. Most of the CqTH genes were relatively highly expressed in different tissues 2 h after exposure to high and low temperatures, osmotic pressure, and salt stress conditions. However, most of the CqTH genes were highly expressed in the different plant organ only after 24 h of exposure to flooding and UV radiation (Fig. 6, Table S6b). The 10 CqTH genes exhibited different expression patterns in specific tissues under various stress conditions. Particularly, most of the genes were upregulated in the leaves, with some expressed in the stems and roots. However, *CqTH18, CqTH19, CqTH25, CqTH28,* and *CqTH42* were upregulated in the stems after flooding for 24 h. Moreover, *CqTH18* was upregulated in the stems after low temperature, high temperature, flooding, and osmotic treatments for 24 h and in the roots after salt treatment for 24 h. Across the different treatments, the 10 genes were consistently expressed in specific tissues. For example, all genes were upregulated in UV-radiated leaves after 24 h, with *CqTH36* reaching high expression levels after only 2 h of treatment. Under salt stress, *CqTH02, CqTH19, CqTH25,* and *CqTH28* were significantly downregulated in different tissues. Additionally, *CqTH02* was also significantly downregulated in different tissues after exposure to low temperature, high temperature, and flooding treatments. Among the 10 genes, *CqTH36* showed the highest expression in the leaves after only 2 h of abiotic stress treatment.

**Fig. 7:**
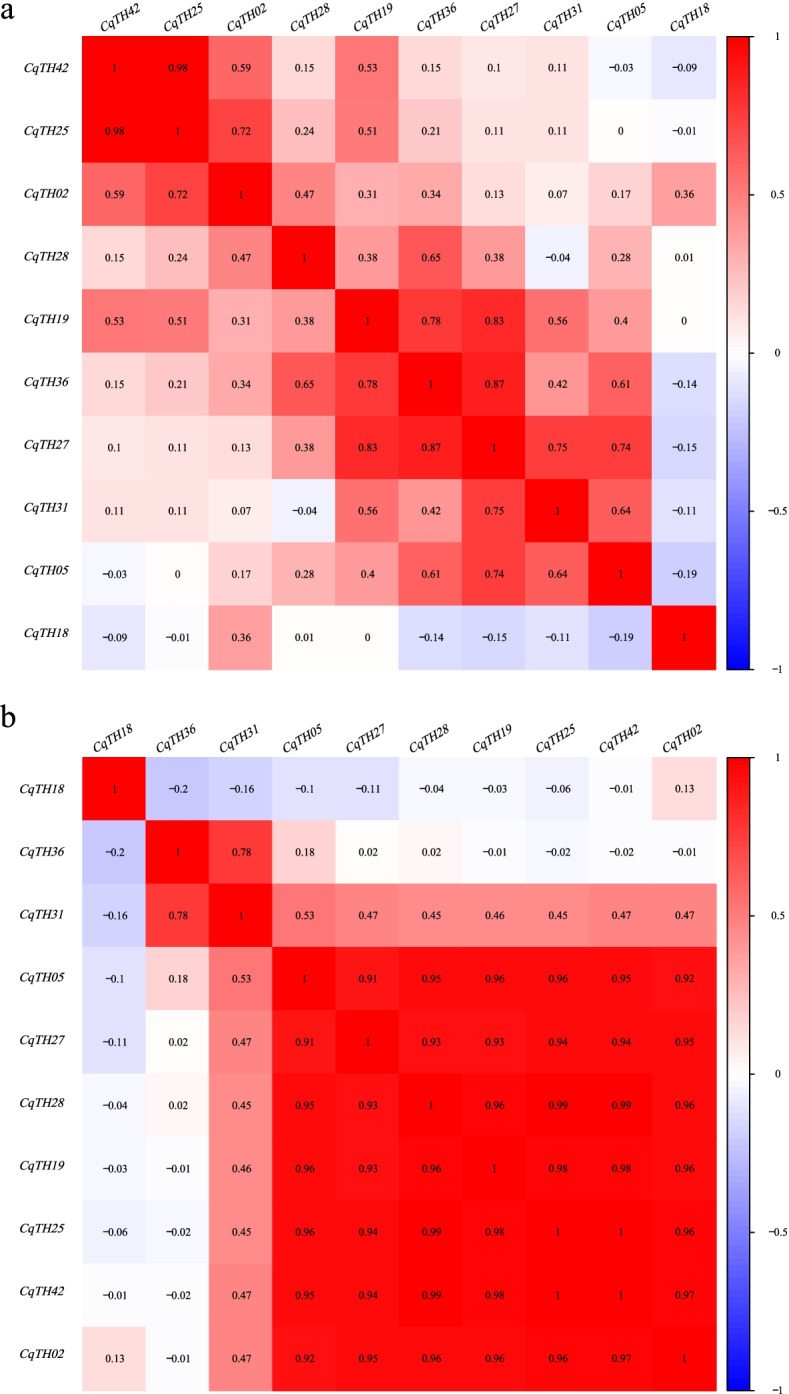
**a** Correlation coefficient diagram of relative expression of 10 CqTH genes in roots, stems and leaves of quinoa seedlings treated with 2 h. Red squares or positive number: positive correlation, light blue squares or negative number: negative correlation. The significance level was 0.01. **b** Correlation coefficient diagram of relative expression of 10 CqTH genes in roots, stems and leaves of quinoa seedlings treated with 24 h. Red squares or positive number: positive correlation, light blue squares or negative number: negative correlation. The significance level was 0.01

Furthermore, we examined the correlation between the expression patterns of the 10 CqTH genes. There was no significant correlation between the expression levels of most genes after 2 h of abiotic stress treatment, with only a few genes highly associated with each other. Specifically, there was no correlation between *CqTH42* and *CqTH02* and the other genes, except *CqTH25*, whereas a negative correlation was observed between *CqTH18* and three other genes, including *CqTH36*, *CqTH27,* and *CqTH05* (Fig. 7a). After 24 h of treatment, no correlation was detected between *CqTH18*, *CqTH36*, and *CqTH31*, whereas a significantly positive correlation was observed between *CqTH05*, *CqTH27*, *CqTH28*, *CqTH19*, *CqTH25*, *CqTH42*, and *CqTH02* (Fig. 7b).

### Expression patterns of CqTH genes at different periods after flowering

**Fig. 8:**
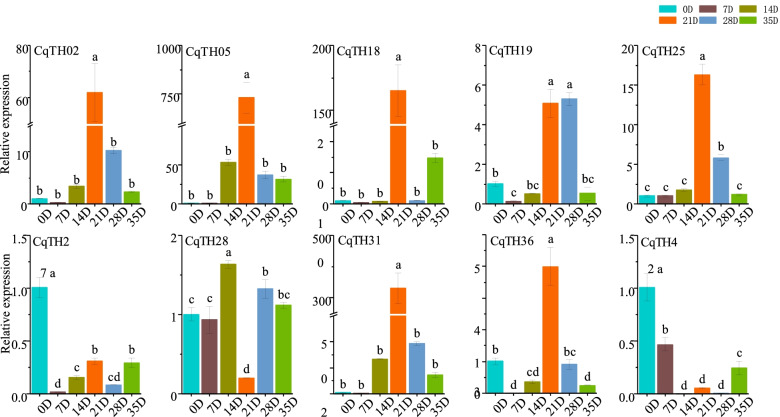
The expression pattern of 10 CqTH genes at different periods after flowering. 0D, 7D, 14D, 21D, 28D and 35D respectively represent the 0, 7th, 14th, 21st, 28th and 35th day after flowering

Subsequently, the expression profiles of the 10 CqTH genes at various periods of quinoa achenes development was examined. There was an increase in the expression of seven genes, including *CqTH02*, *CqTH05*, *CqTH18*, *CqTH19*, *CqTH25*, *CqTH31*, and *CqTH36*, 21 d after flowering, after which the expression levels gradually decreased. Among the genes, *CqTH05* had a > 700-fold change in expression. Additionally, *CqTH19* expression was significantly upregulated 28 d after anthesis, whereas *CqTH28* expression was upregulated only 14 days after anthesis. Moreover, there was significant downregulation in *CqTH42* and *CqTH27* expression after anthesis (Fig. 8, Table S6c).

### CqTH genes expression patterns in response to phytohormone treatments at various stages after flowering

**Fig. 9:**
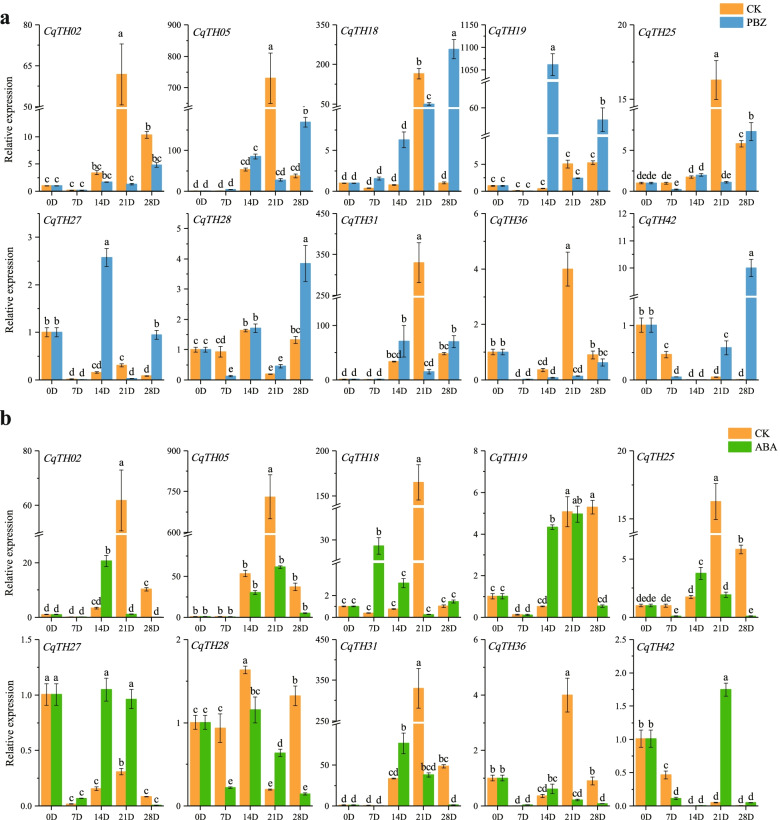
The expression patterns of 10 CqTH genes at different periods after flowering in response to treatment with the plant growth regulator and the phytohormone. CK: not sprayed (control), PBZ: sprayed with Paclobutrazol, ABA: sprayed with Abscisic acid. 0D,7D,14D,21D,28D indicate day 0, day 7, day 14, day 21, day 28 after spraying respectively. **a** The expression patterns of 10 CqTH genes at different periods after flowering in response to treatment with PBZ. **b** The expression patterns of 10 CqTH genes at different periods after flowering in response to treatment with ABA

To investigate the expression patterns of the CqTH genes in response to phytohormone treatment, we sprayed the flowers of the plants with paclobutrazol (PBZ) and abscisic acid (ABA) during the flowering stage and collected samples at weekly intervals. PBZ-treated plants had significantly higher expression of *CqTH05*, *CqTH18*, *CqTH19*, *CqTH25*, *CqTH27*, *CqTH28*, *CqTH31*, and *CqTH42* than plants in the control group at different periods. Specifically, there was a 200-fold increase in *CqTH05* and *CqTH18* expression 28 d post-treatment compared with that of the control plants. Additionally, *CqTH19* expression was > 1000-fold higher than that of plants in the control group 14 d post-treatment. In contrast, PBZ-treated plants had lower expression levels of *CqTH02* and *CqTH36* than plants in the control group at several periods after the PBZ treatment (Fig. 9a, Table S6d). ABA treatment significantly decreased the relative profiles of several genes at several periods compared with the control group, with a decrease in *CqTH05* expression from 750-fold to 50-fold after 21 d of treatment. In contrast, ABA treatment significantly increased the expression patterns of *CqTH02*, *CqTH18*, *CqTH19*, *CqTH25*, *CqTH27*, and *CqTH31* after 14 d compared with the control group (Fig. 9b, Table S6d).

## Discussion

*C. quinoa* is a drought, cold, and salt tolerant crop plant that is grown mainly for its edible seeds, which are rich in protein, fiber, and bioactive compounds. However, whole-genome studies on the quinoa trihelix gene family have not been published. In this study, a total of 47 trihelix genes were identified in *C. quinoa* genome, which is in agreement with the studies on tomato, sorghum, and rice [9, 21, 22]. The CqTH genes account for 0.11% of the total genes in the *C. quinoa* genome [18], which is similar to *A. thaliana* (0.11%) [23], *G. max* (0.14%) [24], and *O. sativa* (0.10%) [25] and higher than that of *S. lycopersicum* (0.05%) [26], *Chrysanthemum* (0.04%) [27], *Triticum* (0.08%) [28], and *F. tataricum* (0.06%) [29]. Trihelix genes were initially grouped into three separate subfamilies, namely GTα, GTβ, and GTγ [16]. However, Kaplan-Levy et al. classified rice (*O. sativa*) and *Arabidopsis* trihelix genes into five subfamilies: GT1, GT2, SH4, SIP1, and GTγ [19]. In the present study, phylogenetic analysis showed that the CqTH genes were classified into five subfamilies: GT1, GT2, SH4, SIP1, and GTγ subfamilies. Moreover, at least one CqTH protein was detected in each subgroup of AtTH and BvTH proteins [30], indicating that the differentiation of the trihelix TF family occurred prior to monocot–dicot divergence.

Additionally, the results of the motif composition and gene structure analysis of the 47 CqTH genes were consistent with the phylogenetic classification of the genes. Most motifs in the same family possess considerable similarities, indicating that the conserved motifs may be necessary for the normal function of specific CqTH proteins. Genes with similar motifs may have been generated by gene duplication events within the same population, which was similar to findings in chrysanthemum [31]. Particularly, the homology between the other subfamilies was considerably lower than that between the GT1 and GT2 subfamilies [32]. Gene duplication, which is one important evolutionary mechanisms for generating new genes, directly influences the adaptation of species to changing environments [33, 34]. Generally, tandem and segmental duplications are the most notable drivers of gene family expansion [33], and thus, are important contributors to protein enrichment and gene evolution [35]. Interestingly, *C. quinoa* possessed less trihelix genes than *G. max* (71), *P. trichocarpa* (56), and *B. napus* (52) [36–38], but more than *Chrysanthemum* (20), *F. tataricum* (31), and *S. lycopersicum* (36) [9, 39, 40]. This discrepancy could be attributed to whole-genome duplication events that may have occurred after species divergence from the earliest land plants. Therefore, we speculated that these fragment duplication events facilitated the differentiation and evolution of several CqTH genes, similar to a previous report in *P. trichocarpa* [37]. Similarly, 6 (29.3%), 13 (36.6%), and 7 (35.0%) duplicated gene pairs have been detected in 41 rice, 71 soybean, and 40 sorghum trihelix genes, respectively [21, 22, 41]. However, the absence of CqTH genes on Chr8 and Chr11 indicates that gene deletion events may have also occurred during the evolutionary process [42]. The loss of CqTH genes could be attributed to dynamic changes during fragment duplication [37]. In the present study, segmental duplication events were the major drivers of the increased number of trihelix genes in *C. quinoa*, accounting for 40 trihelix genes (85%) in the species. Further analysis revealed that these trihelix genes were linked within subfamilies, and certain trihelix genes might be created via duplication events.

Furthermore, we investigated the exon/intron architecture of the 47 CqTH genes and found that the count of exons varied from 1 to 15 (Fig. 2A, B and Table S1), which is similar to findings in rice and sorghum. Additionally, the proportion of CqTH genes without introns (5, 10.6%) was lower than that of rice (18, 43.9%) and sorghum (15, 37.5%) [21, 22]. Interestingly, the majority of intron-free genes were found in the GTγ and SIP1 subfamilies, which was previously observed in *A. thaliana* [23]. Introns can increase gene length and enhance the likelihood of genetic recombination, both of which are favorable for species evolution. Moreover, intron-free genes are more responsive to environmental changes [43]. The findings of the present study showed that the GTγ subfamily had the least amount of exons, while the GT1 subfamily had the highest amount of exons, which is consistent with findings of studies done on wheat [44] and buckwheat [39]. Similar to the reports on chrysanthemum [40], wheat [44], and *Medicago truncatula* [45], the motif compositions of the SIP1 subfamily were distinct from those of other subfamilies. These results indicate that SIP1 subfamily genes may play important and diversified roles in *C. quinoa*.

Since the trihelix TF family has been implicated in the formation of plant organs in previous studies [39], we assessed the expression levels of CqTH genes in the stems, roots, leaves, flowers, and achenes of *C. quinoa*. Several selected CqTH genes showed considerable differential expression in the different organs (Fig. 5a). Notably, *CqTH02*, which belongs to the GT2 subfamily, had the highest expression levels in the leaves and achenes, similar to the expression pattern of its homolog *AT5G03680.1* in *A. thaliana*. Moreover, this gene is involved in regulating leaf structure creation and achenes development in *A. thaliana* [46]. However, *CqTH02* expression was low in roots and stems, which may be related to its subcellular localization to the mitochondria. *CqTH31*, a GT1 subfamily member, was also markedly expressed in the leaves and achenes, which is similar to the expression pattern of its homolog *AT1G13450* in *A. thaliana* [47]. Furthermore, the achenes had considerably higher expression of *CqTH05, CqTH18, CqTH25*, and *CqTH36* than the roots, stems, leaves, and flowers. It could be speculated that these tissue-specific trihelix genes are important in the development and differentiation of the relevant organs [48]; however, additional research is required to confirm their functions. Additionally, several CqTH genes, such as *CqTH25* and *CqTH42*, exhibited significant positive correlations with each other (Fig. 7a and b). *CqTH25*, *CqTH42*, and *AtTH13* are all members of the SIP1 subgroup and share similar motif components (Fig. 2). Notably, the expression patterns of *CqTH25* and *CqTH42* in various organs under different abiotic stresses were similar to that of *AtTH13* [48]. However, further studies are necessary to confirm the putative association between these genes and their response to abiotic stress in different organs. The significant positive correlations observed between the expression profiles of CqTH genes suggest that they may play a synergistic role in organ development and abiotic stress response (Fig. 5b).

The expression patterns of the 10 CqTH genes in seedlings exposed to six different stressors were determined to elucidate the role of the trihelix TF family in adaptation to stress (Fig. 6). There was a considerable increase in the expression levels of the selected genes in the leaves after 24 h exposure to UV radiation, indicating that they may be involved in plant protection against UV damage, especially at high altitudes. Notably, *CqTH28* also displayed high expression levels in UV-radiated leaves. As members of the GT2 subfamily, the motif composition between *AtTH26* and *CqTH28* is comparable. Previous research showed that *AtTH26* (*At5G28300*) was activated in *Arabidopsis* inflorescence and leaves to improve salt, cold, drought, and ABA stress [49]. Additionally, *ShCIGT*, a cold-inducible gene identified in wild tomato, has been shown to promote abiotic stress resistance [50]. Similarly, *CqTH36* expression was considerably upregulated under all abiotic stress conditions, indicating that it may be involved in stress resistance in *C. quinoa*. In vitro and yeast system studies demonstrated that the GT1 cis-element interacts with the GT1-like TF *AtGT-3b* in *Arabidopsis*. Interestingly, *AtGT-3b* transcription was upregulated within 30 min following the salt treatment, indicating that it is more resistant to salt stress [51]. *GTL1* functions as a transcriptional suppressor for the *STOMATAL DENSITY AND DISTRIBUTION 1* (*SDD1*) promoter, which influences stomatal development and transpiration [49, 51, 52]. Notably, *CqTH18* was significantly expressed in different tissues in response to all stressors, indicating that certain novel evolutionary pathways in quinoa may be the product of environmental adaptations to different stressors. Several studies have shown that TH-TFs are not only in abiotic stress response but also in disease resistance [14]. For instance, there was a rapid increase in the expression of the GT1-like gene *RML1* in rice seedlings infected with *Magnaporthe grisea* to minimize pathogen damage [13]. In *Arabidopsis*, *GTL1* regulates salicylic acid homeostasis and acts as a bacteria-induced immunological factor associated with the MPK4 pathway [53]. The results of the present study showed that there was a considerable decrease in the expression of *CqTH02*, which belongs to the GT2 subfamily, in various organs under six different stress conditions, indicating that the gene is negatively regulated in response to abiotic stresses. Similarly, light stress suppressed the expression of the GT2 subfamily gene *PHYA* in rice [20]. Overall, there were significant differences in the expression patterns of the CqTH genes belonging to the five subfamilies depending on the stressor, indicating that individual genes may be involved in distinct physiological activities. These findings suggest that the trihelix gene family may play crucial roles in *C. quinoa* tissue formation and abiotic stress response; however, further studies are necessary to validate their functions.

Promoter cis-elements play important roles in biotic and abiotic stress responses in plants [54]. In the present study, most promoter regions of CqTH genes contained phytohormone, biotic, and abiotic stress responsiveness cis-elements, including abscisic acid-, MeJA-, gibberellin-, low-temperature-, salicylic acid-, defense and stress-, light-, and auxin-responsiveness elements, drought-inducibility element, enhancer-like element involved in anoxic specific inducibility, indicating they play important functions under different stresses. Notably, the higher expression of *CqTH36* under the six abiotic stresses was associated with the presence of multiple cis-acting elements in its promoter region.

Interestingly, *CqTH27* and *CqTH42* were highly upregulated in the flowers of *C. quinoa* during flowering but were poorly expressed in the seeds, which is similar to the expression of the *PETAL LOSS* gene in *Arabidopsis* [55]. *PETAL LOSS* gene is involved in regulating flower development in *Arabidopsis*, indicating that *CqTH27* and *CqTH42* may also be involved in regulating flower development in *C. quinoa*. Furthermore, PBZ treatment significantly upregulated most CqTH genes, whereas ABA treatment significantly downregulated CqTH genes. This discrepancy was attributed to differences in the physiological functions of the two hormones [56, 57].

## Conclusions

In summary, this is study is the first genome-wide analysis of the trihelix gene family in *C. quinoa*. A total of 47 trihelix genes distributed across 17 chromosomes were identified in *C. quinoa* in this study and classified into five subfamilies. Additionally, we identified 23 pairs of segmental duplications in the CqTH gene family, demonstrating that the trihelix gene evolution in quinoa was mostly driven by gene duplication events. Based on the expression profiles of the CqTH genes in different plant tissues under six abiotic stress conditions, at different stages of achene development, and in reaction to phytohormone treatments, some key candidate genes were screened out. For instance, we observed the significant differential expression of several CqTH genes, specifically *CqTH31* during quinoa seed production, *CqTH36* under abiotic stress, *CqTH05* at 21 d after flowering, and *CqTH19* at 14 d after PBZ treatment. Overall, the findings of the present study could serve as a propeller for further studies on the functions of CqTH genes and provide putative genes for the breeding of stress-resistant quinoa varieties.

## Methods

### Identification of *C. quinoa* trihelix genes

The whole *C. quinoa* genome sequence (Cq PI614886 genome V1 pseudomolecule) was downloaded from the ChenopodiumDB database (https://www.cbrc.kaust.edu.sa/chenopodiumdb/) [18], and the trihelix family members were identified by two BLASTp searches [58]. First, all.

possible trihelix proteins with score value ≥100 and e value ≤1^− 10^ were identified from the *C. quinoa* genome, referring to trihelix protein sequences of *A. thaliana* by BLASTp search. Thereafter, the hidden Markov model (HMM) profile corresponding to the trihelix domain (PF13837) was obtained from the Pfam protein family database (http://pfam.sanger.ac.uk/) [59]. Candidate proteins containing the trihelix motifs were screened using HMMER3.0 (http://plants.ensembl.org/hmmer/index.html) at a cutoff of 0.01 [60] and SMART (http://smart.embl-heidelberg.de/) [61]. Information on basic features of the candidate CqTH proteins, including coding sequence lengths, isoelectric points, molecular weight, and subcellular localization, was obtained from the ExPasy website (http://web.expasy.org/protparam/) [62].

### Characterization of trihelix gene structure

Multiple sequence alignments of the CqTH proteins were generated using ClustalW with default parameters [63]. The inferred amino acid sequences in the trihelix domains were manually altered using MEGA 7.0 [64]. The exon/intron structures of the CqTH genes were generated using TBtools [65]. The conserved motifs in the CqTH proteins were identified and compared to detect the motif differences using MEME (http://meme-suite.org/tools/meme), an online search software [66]. The largest number of motifs and motif size were set to 10 and 15–50 amino acid residues, respectively [67–69].

### Analysis of cis-acting elements in the promoter region of the CqTH gene family

To further investigate the function of the CqTH gene family in the *C. quinoa* genome, the 1500 bp region upstream of the CqTH genes was extracted from the *C. quinoa* genome data file as the promoter sequence using TBtools [65] and analyzed using the online software PlantCARE (http://bioinformatics.psb.ugent.be/webtools/plantcare/html/) [70]. Cis-acting elements related to stress response, hormone response, and light response were selected. Finally, graphical visualization was performed using TBtools.

### Determination of chromosomal location and gene duplication events

All CqTH genes were mapped to the *C. quinoa* Willd. chromosomes based on physical location information from the database of the *C. quinoa* genome to determine their physical locations in the genome using Circos [71]. The detection and study of the gene duplication events were performed using multiple collinear scanning (MCScanX) toolkits with the default settings [72]. The homology of trihelix genes between *C. quinoa* Willd. and other representative plants, including *S. bicolor, O. sativa, B. rapa, S. lycopersicum, A. thaliana,* and *S. tuberosum*, was analyzed using Dual Synteny Plotter (https://github.com/CJ-Chen/TBtools) [65]. Non-synonymous (ka) and synonymous (ks) substitutions of each duplicated trihelix gene were calculated using Ka/Ks-Calculator 2.0 [73].

### Phylogenetic analysis and classification of trihelix gene family

Multiple sequence alignments of the AtTH, BvTH, and CqTH proteins were performed to generate an unrooted phylogenetic tree using the NJ method in MEGA 7.0 software with 1000 bootstrap repetitions with default parameters [64]. The phylogenetic trees were constructed using full-length amino acid sequences of trihelix proteins from *C. quinoa, A. thaliana, V. vinifera, S. lycopersicum, B. distachyon, O. sativa,* and *Z. mays* (Table S1). The trihelix protein sequences were retrieved from the UniProt database (UniProt https://www.uniprot.org/) [74], and the discovered CqTH genes were classified into distinct subfamilies.

### Plant materials, growth conditions, and experimental design

The “Qingli No. 1” variety of *C. quinoa*, which was bred by the Qinghai Academy of Agricultural Sciences and validated by the Crop Variety Validation Committee of Qinghai Province in 2016, was used for this study. This variety was introduced by the Institute of Upland Food Crops, Guizhou Academy of Agricultural Sciences, identified by Prof. Liyi Zhang, and preserved in the germplasm repository with the conservation number GL078. The use of this plant material was licensed and approved by the Guizhou Academy of Agricultural Sciences and the Qinghai Academy of Agricultural Sciences. The *C. quinoa* plants used in this study were grown in the Guizhou University greenhouse from May–September 2021. The plants were cultivated in pots filled with soil and vermiculite (1:1) in a growth chamber at 16 h/25 °C during the day and 8 h/20 °C at night, with a relative humidity of 75%. At the five-leaf stage, five healthy plants were harvested, and the stems, roots, and leaves were sampled. The plant parts were promptly frozen in liquid nitrogen and kept at − 80 °C for further analysis. The expression patterns of selected CqTH genes in the different plant parts of 24-day-old quinoa plants under various abiotic stress conditions for 2 h and 24 h were determined by qPCR. Specifically, the seedlings were exposed to salt (400 mM NaCl) [75], flooding (whole plant), osmotic pressure (30% PEG6000) [76], UV light (70 W/cm^2^, 220 V, 30 W), and high (40 °C) and low (4 °C) temperature stress (under 80% light, 16 h during the day and 8 h at night, and 75% humidity). Each stress experiment was performed using five replicates. Quinoa plants used for later sampling were planted in the Guizhou Academy of Agricultural Sciences’ experimental plot, and the culture and management practices were consistent with field practices. Samples of different tissues for qPCR were obtained from the roots, stems, leaves, and flowers of five quinoa plants of uniform length at flowering, and the fruits were obtained at maturity. During flowering, six plants with uniform growth were selected for mixed sampling at 0, 7, 14, 21, 28, and 35 days after flowering. Twelve additional plants were designated for phytohormone treatments, in which half were sprayed with 25 mL/L of ABA solution and the other half were sprayed with 250 mL/L PBZ. Mixed samples obtained at 7, 14, 21, and 28 days after flowering were immediately placed in liquid nitrogen and transported to the laboratory for storage at − 80 °C.

### Total RNA extraction, cDNA synthesis, and qPCR

Total RNA was isolated from each organ using the RNAprep Pure Plant Plus Kit (DP441; TIANGEN Biotech Co. Ltd., Beijing, China) and reverse-transcribed to generate cDNAs. qPCR was performed using qPCR SYBR Green Premix (Vazyme, Nanjing, China) and CFX96 Touch Real-time PCR Detection System (Bio-Rad Laboratories, Hercules, CA, USA). The primer sequences for the ten CqTH genes were generated using Primer Premier 5.0 software [77] (Table S7). We used the *GAPDH* gene as the internal control because of its consistent expression across developmental stages in most plant organs [78]. qPCR was performed using three biological replicates and three technical replicates per sample. The relative expression of the target genes was determined using the 2^-ΔΔCT^ method [79].

### Statistical analysis

Data acquired in this study were subjected to ANOVA using SPSS software (IBM SPSS, Armonk, NY, USA). Mean values were compared and performed using Fisher’s least significant difference (LSD) test, and means were considered significant at *p* < 0.05. Histograms were generated using Origin 8.0 (OriginLab Corporation, Northampton, MA, USA). Tajima’s D neutrality test was performed using MEGA 7.0 [64, 80].

## Supplementary Information

**Additional file 1: Table S1.** The physicochemical properties, cds and protein sequences of the 47 CqTH genes identified in this study.

**Additional file 2: Table S2.** Analysis and distribution of the conserved motifs in CqTH proteins.

**Additional file 3: Figure S1.** Distribution of cis-acting elements in the promoter region of the CqTH gene family.

**Additional file 4: Figure S2.** Schematic representation of the chromosomal distribution of the *C.quinoa* trihelix genes.

**Additional file 5: Table S3.** The 23 pairs of segmental duplicates in CqTH genes.

**Additional file 6: Figure S3.** Phylogenetic relationship and motif composition of the trihelix proteins from *C.quinoa* with five different plant species.

**Additional file 7: Table S4.** One-to-one orthologous gene relationships between *C.quinoa* and other plants.

**Additional file 8: Table S5.** Results of Tajima’s D neutrality test of the CqTH genes family.

**Additional file 9: Table S6.** Relative expression data and ANOVA results for 10 selected CqTH genes.

**Additional file 10: Table S7.** Primer sequences of 10 selected CqTH genes and reference genes for qPCR.

## Data Availability

The complete information on *C. quinoa* genome sequence (Cq_PI614886_genome_V1_pseudomolecule) was obtained from the ChenopodiumDB website (https://www.cbrc.kaust.edu.sa/chenopodiumdb/). All data generated and analyzed during this study are included in this published article and its supplementary information files.

## Abbreviations

AtTH: *Arabidopsis thaliana* trihelix
BvTH: *Beta vulgaris* trihelix
chlo: Chloroplast
CqTH: *Chenopodium quinoa* trihelix
cyto: Cytoplasm
E.R.: Extracellular
HMM: Hidden Markov Model
LG: Linkage group
mito: Mitochondria
nucl: Nucleus
qPCR: Real-time quantitative polymerase chain reaction
TF: Transcription factor
TH: Trihelix

## References

[1] Jin JP, Tian F, Yang DC, Meng YQ, Kong L, Luo JC, et al. PlantTFDB 4.0: toward a central hub for transcription factors and regulatory interactions in plants. Nucleic Acids Res. 2017;45(D1):D1040–5. 10.1093/nar/gkw982 PMC5210657 27924042

[2] Lindemose S, O'Shea C, Jensen MK, Skriver K (2013). Structure, function and networks of transcription factors involved in abiotic stress responses. Int J Mol Sci.

[3] Riechmann JL, Heard J, Martin G, Reuber L, Jiang C, Keddie J, Adam L, Pineda O, Ratcliffe OJ, Samaha RR (2000). *Arabidopsis* transcription factors: genome-wide comparative analysis among eukaryotes. Science (New York, NY).

[4] Green PJ, Yong MH, Cuozzo M, Kano-Murakami Y, Silverstein P, Chua NH (1988). Binding site requirements for pea nuclear protein factor GT-1 correlate with sequences required for light-dependent transcriptional activation of the *rbcS-3A* gene. EMBO J.

[5] Green PJ, Kay SA, Chua NH (1987). Sequence-specific interactions of a pea nuclear factor with light-responsive elements upstream of the *rbcS-3A* gene. EMBO J.

[6] Nagano Y (2000). Several features of the GT-factor trihelix domain resemble those of the Myb DNA-binding domain. Plant Physiol.

[7] Yao Q, Xin M, Guanghui Y, Qi W, Liang W, Lingrang K, Wook K, Wei WH (2014). Evolutionary history of trihelix family and their functional diversification. Narnia.

[8] Gao MJ, Lydiate DJ, Li X, Lui H, Gjetvaj B, Hegedus DD, Rozwadowski K (2009). Repression of seed maturation genes by a Trihelix transcriptional repressor in *Arabidopsis* seedlings. Plant Cell.

[9] Yu C, Cai X, Ye Z, Li H (2015). Genome-wide identification and expression profiling analysis of trihelix gene family in tomato. Biochem Biophys Res Commun.

[10] Zheng X, Liu HP, Ji HT, Wang YN, Dong BD, Qiao YZ, Liu MY, Li X (2016). The wheat GT factor *TaGT2L1D* negatively regulates drought tolerance and plant development. Sci Rep-Uk.

[11] Shibata M, Breuer C, Kawamura A, Clark NM, Rymen B, Braidwood L, Morohashi K, Busch W, Benfey PN, Sozzani R (2018). *GTL1* and *DF1* regulate root hair growth through transcriptional repression of *ROOT HAIR DEFECTIVE 6-LIKE 4* in *Arabidopsis*. Development.

[12] Murata J, Takase H, Hiratsuka K (2002). Characterization of a novel GT-box binding protein from Arabidopsis. Plant Biotechnol.

[13] Wang R, Hong G, Han B (2004). Transcript abundance of *rml1*, encoding a putative GT1-like factor in rice, is up-regulated by Magnaporthe grisea and down-regulated by light. Gene.

[14] Xie Z, Zou H, Lei G, Wei W, Zhou Q, Niu C, Liao Y, Tian A, Ma B, Zhang W (2009). Soybean Trihelix transcription factors *GmGT-2A* and *GmGT-2B* improve plant tolerance to abiotic stresses in transgenic *Arabidopsis*. PLoS One.

[15] Yoo CY, Pence HE, Jin JB, Miura K, Gosney MJ, Hasegawa PM, Mickelbart MV (2010). The *Arabidopsis GTL1* transcription factor regulates water use efficiency and drought tolerance by modulating stomatal density via transrepression of *SDD1*. Plant Cell.

[16] Fang Y, Xie K, Hou X, Hu H, Xiong L (2010). Systematic analysis of GT factor family of rice reveals a novel subfamily involved in stress responses. Mol Gen Genomics.

[17] Zhang D, Wei X, Liu Z, Wu X, Bao C, Sun Y, Su N, Cui J (2021). Transcriptome analysis reveals the molecular mechanism of GABA accumulation during quinoa (*Chenopodium quinoa* Willd.) germination. J Agric Food Chem.

[18] Jarvis DE, Ho YS, Lightfoot DJ, Schmockel SM, Li B, Borm TJA, Ohyanagi H, Mineta K, Michell CT, Saber N (2017). The genome of *Chenopodium quinoa*. Nature.

[19] Kaplan-Levy RN, Brewer PB, Quon T, Smyth DR (2012). The trihelix family of transcription factors--light, stress and development. Trends Plant Sci.

[20] Qin Y, Ma X, Yu GH, Wang Q, Wang L, Kong LR, Kim W, Wang HW (2014). Evolutionary history of Trihelix family and their functional diversification. DNA Res.

[21] Li K, Duan L, Zhang Y, Shi M, Chen S, Yang M, Ding Y, Peng Y, Dong Y, Yang H (2021). Genome-wide identification and expression profile analysis of trihelix transcription factor family genes in response to abiotic stress in sorghum [*Sorghum bicolor* (L.) Moench]. BMC Genomics.

[22] Li J, Zhang M, Sun J, Mao X, Wang J, Wang J, Liu H, Zheng H, Zhen Z, Zhao H (2019). Genome-wide characterization and identification of trihelix transcription factor and expression profiling in response to abiotic stresses in Rice (*Oryza sativa* L.). Int J Mol Sci.

[23] Yasmeen E, Riaz M, Sultan S, Azeem F, Abbas A, Riaz K, Ali MA (2016). Genome-wide analysis of trihelix transcription factor gene family in *Arabidopsis thaliana*. Pak J Agr Sci.

[24] Shen Y, Liu J, Geng H, Zhang J, Liu Y, Zhang H, Xing S, Du J, Ma S, Tian Z (2018). De novo assembly of a Chinese soybean genome. Sci China Life Sci.

[25] Tanaka T, Nishijima R, Teramoto S, Kitomi Y, Hayashi T, Uga Y, Kawakatsu T (2020). De novo genome assembly of the indica rice variety IR64 using linked-read sequencing and nanopore sequencing. G3-Genes Genom Genet.

[26] Takei H, Shirasawa K, Kuwabara K, Toyoda A, Matsuzawa Y, Iioka S, Ariizumi T (2021). De novo genome assembly of two tomato ancestors, *Solanum pimpinellifolium* and *Solanum lycopersicum* var. cerasiforme, by long-read sequencing. DNA Res.

[27] Song C, Liu YF, Song AP, Dong GQ, Zhao HB, Sun W, Ramakrishnan S, Wang Y, Wang SB, Li TZ (2018). The *Chrysanthemum nankingense* genome provides insights into the evolution and diversification of chrysanthemum flowers and medicinal traits. Mol Plant.

[28] Walkowiak S, Gao LL, Monat C, Haberer G, Kassa MT, Brinton J, Ramirez-Gonzalez RH, Kolodziej MC, Delorean E, Thambugala D (2020). Multiple wheat genomes reveal global variation in modern breeding. Nature.

[29] Zhang LJ, Li XX, Ma B, Gao Q, Du HL, Han YH, Li Y, Cao YH, Qi M, Zhu YX (2017). The tartary buckwheat genome provides insights into rutin biosynthesis and abiotic stress tolerance. Mol Plant.

[30] Dohm JC, Minoche AE, Holtgrawe D, Capella-Gutierrez S, Zakrzewski F, Tafer H, Rupp O, Sorensen TR, Stracke R, Reinhardt R (2014). The genome of the recently domesticated crop plant sugar beet (*Beta vulgaris*). Nature.

[31] Song AP, Gao TW, Wu D, Xin JJ, Chen SM, Guan ZY, Wang HB, Jin LL, Chen FD (2016). Transcriptome-wide identification and expression analysis of chrysanthemum *SBP-like* transcription factors. Plant Physiol Biochem.

[32] Kuhn RM, Caspar T, Dehesh K, Quail PH (1993). DNA binding factor GT-2 from *Arabidopsis*. Plant Mol Biol.

[33] Kong H, Landherr LL, Frohlich MW, Leebens-Mack J, Ma H, dePamphilis CW (2007). Patterns of gene duplication in the plant *SKP1* gene family in angiosperms: evidence for multiple mechanisms of rapid gene birth. Plant J.

[34] Moore RC, Purugganan MD (2003). The early stages of duplicate gene evolution. Proc Natl Acad Sci U S A.

[35] Cannon SB, Mitra A, Baumgarten A, Young ND, May G (2004). The roles of segmental and tandem gene duplication in the evolution of large gene families in *Arabidopsis thaliana*. BMC Plant Biol.

[36] Wang WL, Wu P, Liu TK, Ren HB, Li Y, Hou XL (2017). Genome-wide analysis and expression divergence of the trihelix family in *Brassica Rapa*: insight into the evolutionary patterns in plants. Sci Rep-Uk.

[37] Wang Z, Liu Q, Wang H, Zhang H, Xu X, Li C, Yang C (2016). Comprehensive analysis of trihelix genes and their expression under biotic and abiotic stresses in *Populus trichocarpa*. Sci Rep-Uk.

[38] Borges OM, Lauro BN, Graciela C, Carina TZA, Beatriz WS, Helena BZM, Márcia MP (2012). Identification and in silico characterization of soybean trihelix-GT and bHLH transcription factors involved in stress responses. Genet Mol Biol.

[39] Ma ZT, Liu MY, Sun WJ, Huang L, Wu Q, Bu TL, Li CL, Chen H (2019). Genome-wide identification and expression analysis of the trihelix transcription factor family in tartary buckwheat (*Fagopyrum tataricum*). BMC Plant Biol.

[40] Song A, Wu D, Fan Q, Tian C, Chen S, Guan Z, Xin J, Zhao K, Chen F (2016). Transcriptome-wide identification and expression profiling analysis of chrysanthemum trihelix transcription factors. IJMS.

[41] Liu W, Zhang Y, Li W, Lin Y, Wang C, Xu R, Zhang L (2020). Genome-wide characterization and expression analysis of soybean trihelix gene family. PeerJ.

[42] Liu J, Chen N, Chen F, Cai B, Dal Santo S, Tornielli GB, Pezzotti M, Cheng ZM (2014). Genome-wide analysis and expression profile of the bZIP transcription factor gene family in grapevine (*Vitis vinifera*). BMC Genomics.

[43] Shabalina SA, Ogurtsov AY, Spiridonov AN, Novichkov PS, Spiridonov NA, Koonin EV (2010). Distinct patterns of expression and evolution of Intronless and intron-containing mammalian genes. Mol Biol Evol.

[44] Xiao J, Hu R, Gu T, Han JP, Qiu D, Su PP, Feng JL, Chang JL, Yang GX, He GY (2019). Genome-wide identification and expression profiling of trihelix gene family under abiotic stresses in wheat. BMC Genomics.

[45] Liu X, Zhang H, Ma L, Wang Z, Wang K (2020). Genome-wide identification and expression profiling analysis of the trihelix gene family under abiotic stresses in *Medicago truncatula*. Genes (Basel).

[46] Frerichs A, Thoma R, Abdallah AT, Frommolt P, Werr W, Chandler JW (2016). The founder-cell transcriptome in the *Arabidopsis apetala1 cauliflower* inflorescence meristem. BMC Genomics.

[47] Tang XR, Hou AF, Babu M, Nguyen V, Hurtado L, Lu Q, Reyes JC, Wang AM, Keller WA, Harada JJ (2008). The *Arabidopsis* BRAHMA chromatin-remodeling ATPase is involved in repression of seed maturation genes in leaves. Plant Physiol.

[48] Krizek BA, Bantle AT, Heflin JM, Han H, Freese NH, Loraine AE (2021). *AINTEGUMENTA* and *AINTEGUMENTA-LIKE6* directly regulate floral homeotic, growth, and vascular development genes in young *Arabidopsis* flowers. J Exp Bot.

[49] Xi J, Qiu YJ, Du LQ, Poovaiah BW (2012). Plant-specific trihelix transcription factor *AtGT2L* interacts with calcium/calmodulin and responds to cold and salt stresses. Plant Sci.

[50] Yu C, Song L, Song J, Ouyang B, Guo L, Shang L, Wang T, Li H, Zhang J, Ye Z (2018). *ShCIGT*, a Trihelix family gene, mediates cold and drought tolerance by interacting with *SnRK1* in tomato. Plant Sci.

[51] Park HC, Kim ML, Kang YH, Jeon JM, Yoo JH, Kim MC, Park CY, Jeong JC, Moon BC, Lee JH (2004). Pathogen- and NaCl-induced expression of the *SCaM-4* promoter is mediated in part by a GT-1 box that interacts with a GT-1-like transcription factor. Plant Physiol.

[52] Yoo CY, Hasegawa PM, Mickelbart MV (2011). Regulation of stomatal density by the *GTL1* transcription factor for improving water use efficiency. Plant Signal Behav.

[53] Volz R, Kim SK, Mi JN, Mariappan KG, Guo XJ, Bigeard J, Alejandro S, Pflieger D, Rayapuram N, Al-Babili S (2018). The trihelix transcription factor *GT2-like 1* (*GTL1*) promotes salicylic acid metabolism, and regulates bacterial-triggered immunity. PLoS Genet.

[54] Bilas R, Szafran K, Hnatuszko-Konka K, Kononowicz AK (2016). Cis-regulatory elements used to control gene expression in plants. Plant Cell Tiss Org.

[55] Brewer PB, Howles PA, Dorian K, Griffith ME, Ishida T, Kaplan-Levy RN, Kilinc A, Smyth DR (2004). *PETAL LOSS*, a trihelix transcription factor gene, regulates perianth architecture in the *Arabidopsis* flower. Development (Cambridge, England).

[56] Shin K, Lee I, Kim E, Park SK, Soh MS, Lee S (2019). *PACLOBUTRAZOL-RESISTANCE* gene family regulates floral organ growth with unequal genetic redundancy in *Arabidopsis thaliana*. Int J Mol Sci.

[57] Ma Y, Cao J, He J, Chen Q, Li X, Yang Y (2018). Molecular mechanism for the regulation of ABA homeostasis during plant development and stress responses. Int J Mol Sci.

[58] Altschul SF, Madden TL, Schaffer AA, Zhang J, Zhang Z, Miller W, Lipman DJ (1997). Gapped BLAST and PSI-BLAST: a new generation of protein database search programs. Nucleic Acids Res.

[59] Mistry J, Chuguransky S, Williams L, Qureshi M, Salazar GA, Sonnhammer ELL, Tosatto SCE, Paladin L, Raj S, Richardson LJ (2021). Pfam: the protein families database in 2021. Nucleic Acids Res.

[60] Finn RD, Clements J, Eddy SR (2011). HMMER web server: interactive sequence similarity searching. Nucleic Acids Res.

[61] Letunic I, Bork P (2018). 20 years of the SMART protein domain annotation resource. Nucleic Acids Res.

[62] Duvaud S, Gabella C, Lisacek F, Stockinger H, Ioannidis V, Durinx C (2021). Expasy, the Swiss bioinformatics resource portal, as designed by its users. Nucleic Acids Res.

[63] Thompson JD, Gibson TJ, Higgins DG. Multiple sequence alignment using ClustalW and ClustalX. Curr Protoc Bioinformatics. 2003;2-3(1):1–22. 10.1002/0471250953.bi0203s00 18792934

[64] Kumar S, Stecher G, Tamura K (2016). MEGA 7: molecular evolutionary genetics analysis version 7.0 for bigger datasets. Mol Biol Evol.

[65] Chen CJ, Chen H, Zhang Y, Thomas HR, Frank MH, He YH, Xia R (2020). TBtools: an integrative toolkit developed for interactive analyses of big biological data. Mol Plant.

[66] Bailey TL, Boden M, Buske FA, Frith M, Grant CE, Clementi L, Ren JY, Li WW, Noble WS (2009). MEME SUITE: tools for motif discovery and searching. Nucleic Acids Res.

[67] Liu M, Ma Z, Sun W, Huang L, Wu Q, Tang Z, Bu T, Li C, Chen H (2019). Genome-wide analysis of the NAC transcription factor family in Tartary buckwheat (*Fagopyrum tataricum*). BMC Genomics.

[68] Liu M, Ma Z, Wang A, Zheng T, Huang L, Sun W, Zhang Y, Jin W, Zhan J, Cai Y (2018). Genome-wide investigation of the auxin response factor gene family in tartary buckwheat (*Fagopyrum tataricum*). Int J Mol Sci.

[69] Xie T, Chen CJ, Li CH, Liu JR, Liu CY, He YH (2018). Genome-wide investigation of WRKY gene family in pineapple: evolution and expression profiles during development and stress. BMC Genomics.

[70] Lescot M, Dehais P, Thijs G, Marchal K, Moreau Y, Van de Peer Y, Rouze P, Rombauts S (2002). PlantCARE, a database of plant cis-acting regulatory elements and a portal to tools for in silico analysis of promoter sequences. Nucleic Acids Res.

[71] Krzywinski M, Schein J, Birol I, Connors J, Gascoyne R, Horsman D, Jones SJ, Marra MA (2009). Circos: an information aesthetic for comparative genomics. Genome Res.

[72] Wang Y, Tang H, Debarry JD, Tan X, Li J, Wang X, Lee T-h, Jin H, Marler B, Guo H (2012). MCScanX: a toolkit for detection and evolutionary analysis of gene synteny and collinearity. Nucleic Acids Res.

[73] Wang D, Zhang Y, Zhang Z, Zhu J, Yu J (2010). KaKs_Calculator 2.0: a toolkit incorporating gamma-series methods and sliding window strategies. Genomics Proteomics Bioinformatics.

[74] UniProt C (2021). UniProt: the universal protein knowledgebase in 2021. Nucleic Acids Res.

[75] Cai ZQ, Gao Q (2020). Comparative physiological and biochemical mechanisms of salt tolerance in five contrasting highland quinoa cultivars. BMC Plant Biol.

[76] Wang H, Ao P, Yang S, Zou Z, Wang S, Gong M (2015). Molecular cloning and expression analysis of the gene encoding proline dehydrogenase from *Jatropha curcas* L. Appl Biochem Biotechnol.

[77] Zhai ZH, Chen XN, Wang J (2008). Primer design with primer premier 5.0. Northwest Med Educ.

[78] Reddy PS, Reddy DS, Sivasakthi K, Bhatnagar-Mathur P, Vadez V, Sharma KK (2016). Evaluation of Sorghum [*Sorghum bicolor* (L.)] reference genes in various tissues and under abiotic stress conditions for quantitative real-time PCR data normalization. Front Plant Sci.

[79] Livak KJ, Schmittgen TD (2001). Analysis of relative gene expression data using real-time quantitative PCR and the 2^-ΔΔCT^ method. Methods (San Diego, Calif).

[80] Tajima F (1989). Statistical method for testing the neutral mutation hypothesis by DNA polymorphism. Genetics.

