# Supplementary material for: Genome-wide identification, phylogenetic analysis, and expression profiles of trihelix transcription factor family genes in quinoa (Chenopodium quinoa Willd.) under abiotic stress conditions
Source: BMC Genomics. 2022 Jul 10;23:499. doi: 10.1186/s12864-022-08726-y (PMC9271251; doi:10.1186/s12864-022-08726-y)
Supplement: Supplementary file 3 — Additional file 3: Figure S1. Distribution of cis-acting elements in the promoter region of the CqTH gene family. [file 12864_2022_8726_MOESM3_ESM.pdf]

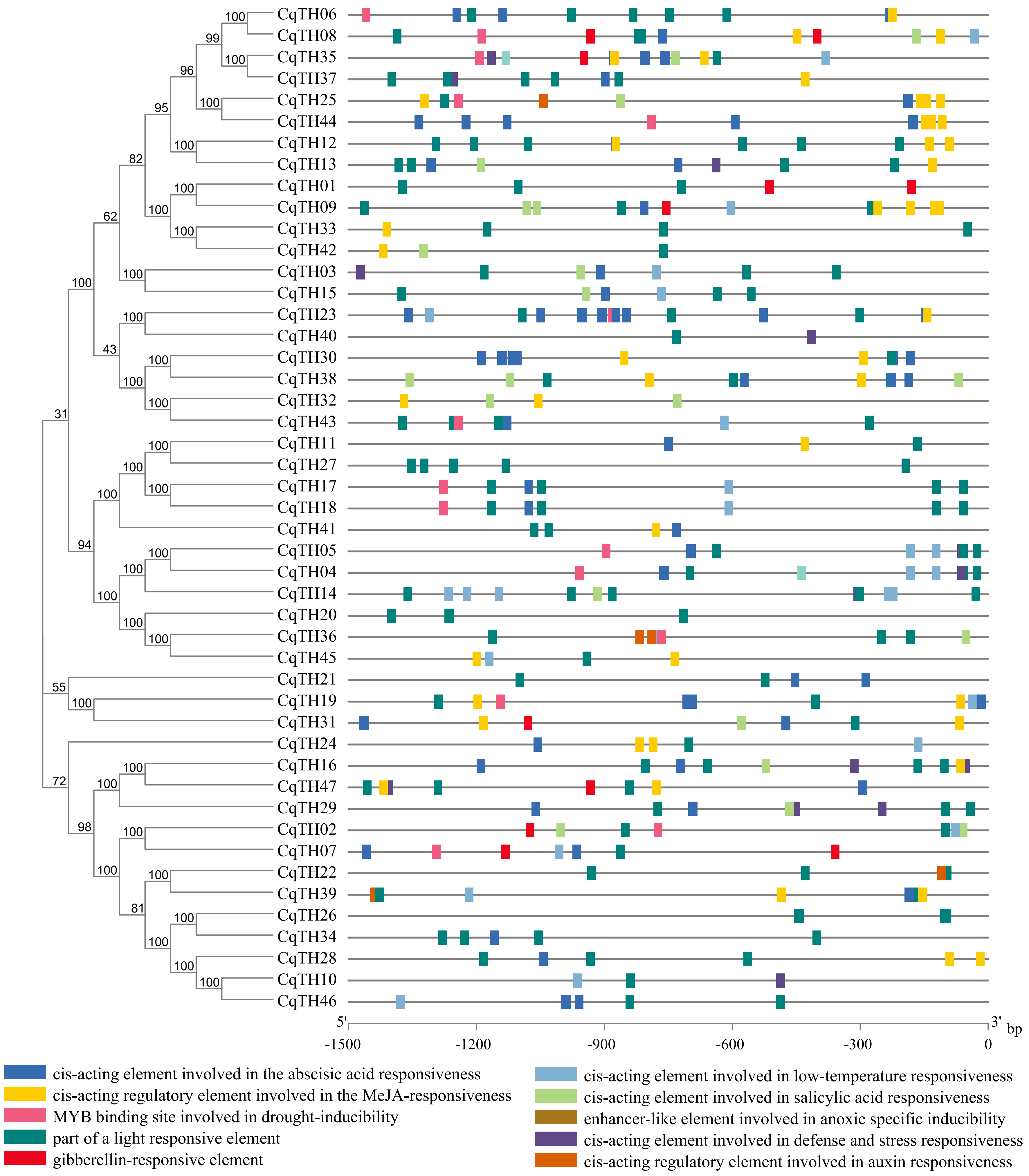

Fig. S1. Distribution of cis-acting elements of CqTH genes. All promoter sequences (-1500 bp) were analysed. The CqTH genes are shown on the left. Scale bar at the base indicates length of promoter sequences. Cis-acting elements and names were presented by different colours.
