# Supplementary material for: Genome-wide identification, phylogenetic analysis, and expression profiles of trihelix transcription factor family genes in quinoa (Chenopodium quinoa Willd.) under abiotic stress conditions
Source: BMC Genomics. 2022 Jul 10;23:499. doi: 10.1186/s12864-022-08726-y (PMC9271251; doi:10.1186/s12864-022-08726-y)
Supplement: Supplementary file 4 — Additional file 4: Figure S2. Schematic representation of the chromosomal distribution of the C.quinoa trihelix genes. [file 12864_2022_8726_MOESM4_ESM.pdf]

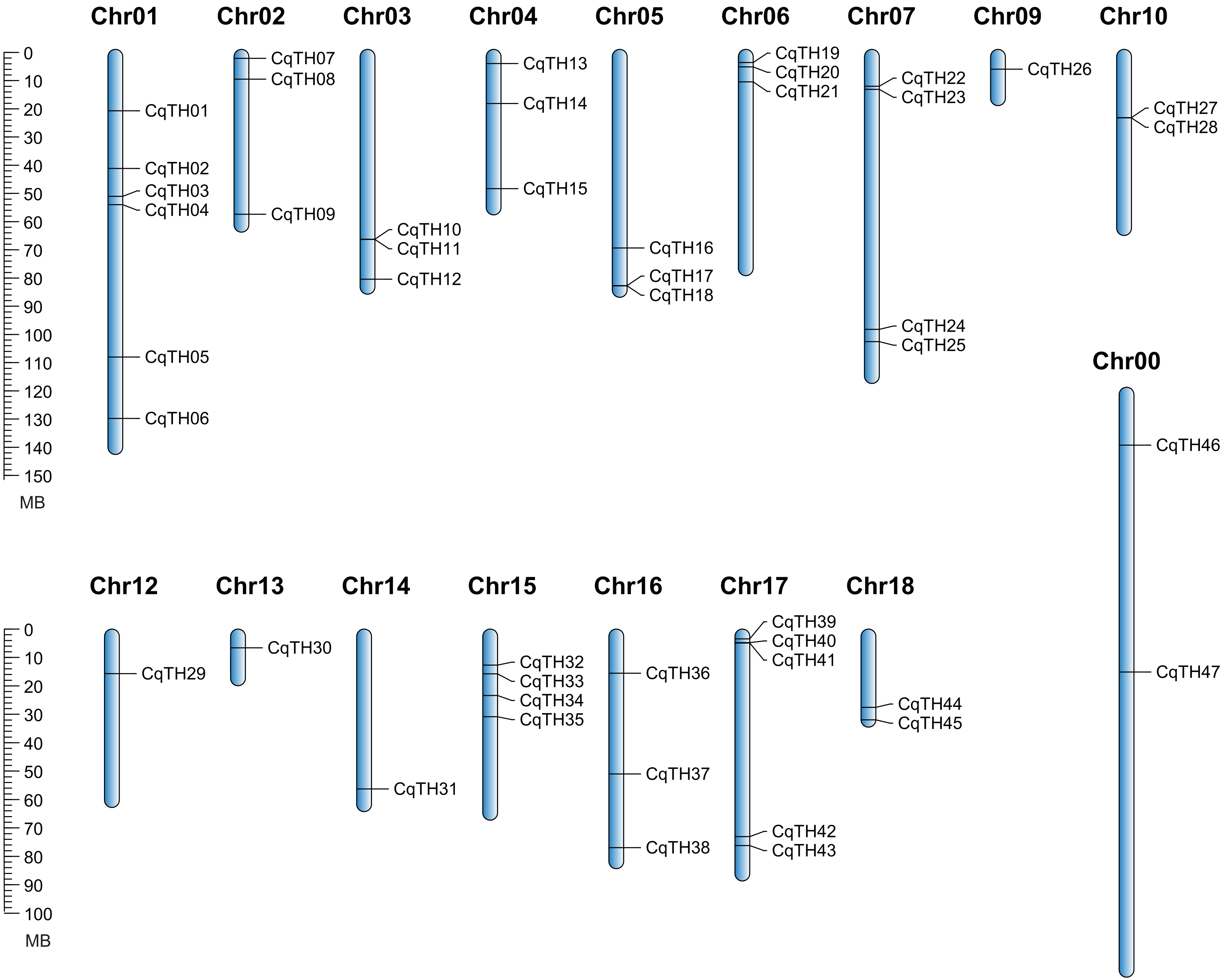

Fig. S2. Schematic representation of the chromosomal distribution of CqTH genes. Vertical bars represent the chromosomes of *C.quinoa*. The chromosome number is indicated at the top of each chromosome. The scale on the left represents chromosome length.
