# Supplementary material for: Genome-wide identification, phylogenetic analysis, and expression profiles of trihelix transcription factor family genes in quinoa (Chenopodium quinoa Willd.) under abiotic stress conditions
Source: BMC Genomics. 2022 Jul 10;23:499. doi: 10.1186/s12864-022-08726-y (PMC9271251; doi:10.1186/s12864-022-08726-y)
Supplement: Supplementary file 6 — Additional file 6: Figure S3. Phylogenetic relationship and motif composition of the trihelix proteins from C.quinoa with five different plant species. [file 12864_2022_8726_MOESM6_ESM.pdf]

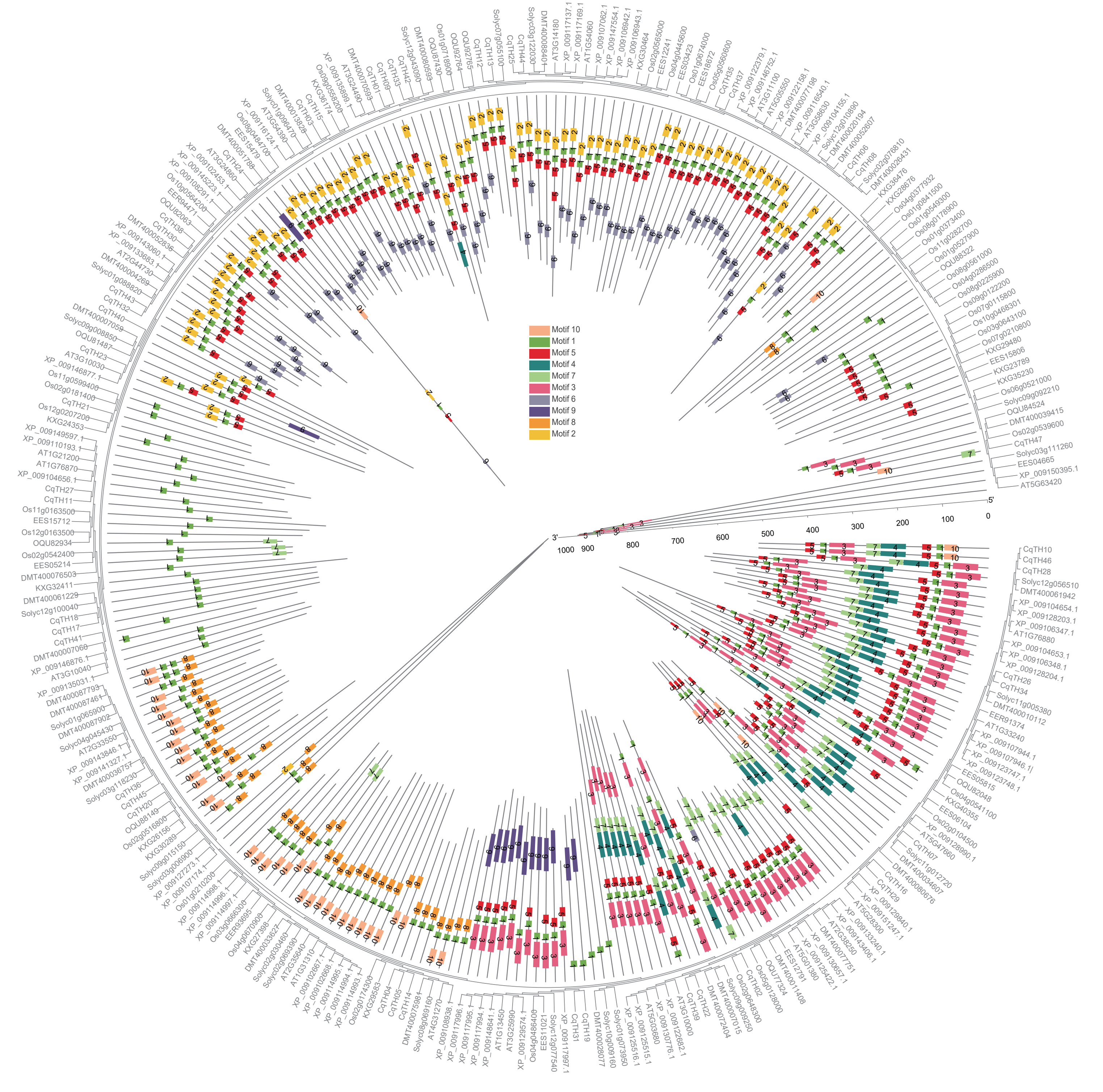

Fig. S3. Phylogenetic relationship and motif composition of the trihelix proteins of *C.quinoa* and six different plant species. Outer panel: An unrooted phylogenetic tree constructed using Geneious R11 with the NJ method. Inner panel: Distribution of the conserved motifs in trihelix proteins. The differently coloured boxes represent different motifs and their positions in each trihelix protein sequence. The sequence information for each motif is provided in Additional File 2: Table S2.
